# CowMesh: a data-mesh architecture to unify dairy industry data for prediction and monitoring

**DOI:** 10.3389/frai.2023.1209507

**Published:** 2023-10-04

**Authors:** Arjun Pakrashi, Duncan Wallace, Brian Mac Namee, Derek Greene, Christophe Guéret

**Affiliations:** ^1^School of Computer Science, University College Dublin, Dublin, Ireland; ^2^Insight Centre for Data Analytics, Dublin, Ireland; ^3^VistaMilk SFI Research Centre, Teagasc Moorepark, Fermoy, Ireland; ^4^Accenture Labs, Dublin, Ireland

**Keywords:** data mesh, data fabric, digital agriculture, dairy industry, decentralized data

## Abstract

Dairy is an economically significant industry that caters to the huge demand for food products in people's lives. To remain profitable, farmers need to manage their farms and the health of the dairy cows in their herds. There are, however, many risks to cow health that can lead to significant challenges to dairy farm management and have the potential to lead to significant losses. Such risks include cow udder infections (i.e., mastitis) and cow lameness. As automation and data recording become more common in the agricultural sector, dairy farms are generating increasing amounts of data. Recently, these data are being used to generate insights into farm and cow health, where the objective is to help farmers manage the health and welfare of dairy cows and reduce losses from cow health issues. Despite the level of data generation on dairy farms, this information is often difficult to access due to a lack of a single, central organization to collect data from individual farms. The prospect of such an organization, however, raises questions about data ownership, with some farmers reluctant to share their farm data for privacy reasons. In this study, we describe a new *data mesh* architecture designed for the dairy industry that focuses on facilitating access to data from farms in a decentralized fashion. This has the benefit of keeping the ownership of data with dairy farmers while bringing data together by providing a common and uniform set of protocols. Furthermore, this architecture will allow secure access to the data by research groups and product development groups, who can plug in new projects and applications built across the data. No similar framework currently exists in the dairy industry, and such a data mesh can help industry stakeholders by bringing the dairy farms of a country together in a decentralized fashion. This not only helps farmers, dairy researchers, and product builders but also facilitates an overview of all dairy farms which can help governments to decide on regulations to improve the dairy industry at a national level.

## 1. Introduction

The dairy industry is experiencing strong global growth (Douphrate et al., 2013; Bhat et al., 2022) accompanied by significant transformations through the increasing adoption of digital technologies (Borchers and Bewley, 2015; Gargiulo et al., 2018; Hansen et al., 2019; Gabriel and Gandorfer, 2023). As the industry enables this growth by producing dairy products more efficiently, it becomes essential to focus on cow health, as well as long-term factors, such as environmental impacts and profitability (Barkema et al., 2015; Bhat et al., 2022). The adoption of digital technologies on dairy farms (Gabriel and Gandorfer, 2023) means that considerably more data are being generated from dairy farms. These data are used to help farmers monitor their farms, make decisions, and achieve their goals around production, profitability, and cattle welfare and also to help administrative bodies to set national and international policies.

Additional availability of farm data also allows advanced statistical and machine learning techniques to be applied to support farm decision-making. Cow health has received significant attention in this regard. Early prediction of ailments in cows can reduce financial losses and improve cattle welfare. Recent adoption of technology in farm and data availability have triggered several advanced predictive analytic studies focused on dairy farms. For example, mastitis (an udder infection that afflicts cows) is one of the top reasons for monetary loss in dairy farms (Yalcin et al., 1999; Petrovski et al., 2006; Viguier et al., 2009), and several data-driven approaches to detecting mastitis are described in the literature (Ebrahimi et al., 2019; Bobbo et al., 2021; Ryan et al., 2021). There are also a number of studies that use data-driven approaches to detect lameness (Shahinfar et al., 2021; Altay and Albayrak Delialioğlu, 2022; Zhou et al., 2022) and ketosis (a metabolic disease in cows; Bauer and Jagusiak, 2022; Wang et al., 2023) using machine learning. As well as cow health, there are other aspects of dairy farming where data-centric advanced systems are being employed, including predicting herbage yield and composition (O'Hara et al., 2021; Albert et al., 2022), and estimating greenhouse gas emission (Chianese et al., 2009; Martin et al., 2017; Kadam and Vijayumar, 2018).

With the digitization of the dairy industry, research and product development are becoming more interdisciplinary. Sophisticated machine learning and statistical systems exploiting the data collected on farms are being developed by research groups in universities and organizations involving farmers, geneticists, computer scientists, and statisticians. A general trend is that several research groups perform research independently, with limited data sharing. This is often due to limited interoperability between data sources, data sharing challenges, and a lack of trust among stakeholders. Nonetheless, sharing the data generated on farms, as well as integrating different products (e.g., analysis, results, and services), has the potential to bring significant economic value to the agriculture industry (Wysel et al., 2021).

Wolfert et al. (2017) identified several major challenges in digital agriculture, which are also present in dairy farming: data ownership, data quality, sustainable integration of data sources, intelligence processing and analytics, business models, and openness of platforms. The World Economic Forum (WEF, 2023) is a non-profit organization that brings together global leaders to address critical issues and promote public-private cooperation, serving as a platform for networking, dialog, and shaping agendas for positive changes. The WEF summarizes the challenges in digital agriculture in three categories: fragmentation, standards, and access. A recent study by Fadul-Pacheco et al. (2022) describes data-oriented issues and demands of dairy farms. It was found that dairy farmers and non-dairy farmers (related to the dairy industry) believed that data sharing is important. However, issues with data ownership and data quality represented a significant area of concern. A significant portion was unsure about the chain of custody of data. Non-farmers (e.g., researchers and organizations without dairy farming expertise) were concerned about the lack of integration of data and, in some cases, not aware of the usefulness of data integration. The issue of trust when sharing data was raised in the study by Jakku et al. (2019), indicating that transparency, trust, and data ownership are major issues in sharing data. This highlights the need for a framework that ensures good data quality, effective data integration, transparent data use and ownership, and effective use of the data to build analytics and predictive systems. This, in turn, demonstrates that there is a requirement for a reliable data sharing framework in the dairy industry context which addresses the above issues.

In this study, we introduce a data mesh architecture, CowMesh, that addresses the challenges in data-driven dairy farming as described above. Specifically, we propose an architecture with a central semantic data product that provides interoperability among other data products and data domains by providing a high-level ontology of dairy farm components and a uniform data access protocol.

The rest of the study is structured as follows. Section 2 discusses related work. The proposed CowMesh architecture is described in detail in Section 3. A series of use cases from the Irish dairy farming context are then presented in Section 4, to show the typical usage of the CowMesh architecture. Key advantages and opportunities for the architecture are discussed in Section 5, and finally, Section 6 concludes the study.

## 2. Related work

According to WEF (2023), the key challenges around data in the agricultural domain can be summarized as follows:

- **Fragmentation**: Data are gathered from a variety of sources (sensors, satellites, etc.) and made available as different topical silos (soil data, seed data, etc.);
- **Standards**: There is no global standard or standardization body facilitating the expression of agricultural data;
- **Access**: Data need to be exchanged and connected in order to deliver value.

However, these challenges are actually not restricted to agriculture and are a larger concern across many different industry sectors. The proposal for making scientific data **F**indable, **A**ccessible, **I**nteroperable, and **R**eusable (FAIR; Wilkinson et al., 2016) is a pragmatic approach toward producing data in a better way. It has been designed with the scientific community in mind, but the principles are more globally applicable. FAIR pushes forward a number of core principles which align largely with the W3C Data on the Web Best Practices (DWBP)^1^ but do not push forward any particular technology stack.

According to both FAIR and DWBP, vocabularies are a pillar of data publication. In the agricultural domain, the thesaurus AGROVOC^2^ from the Food and Agriculture Organization is an important resource that is made available as a SKOS-based ontology (Caracciolo et al., 2013). This vocabulary is essential for describing agricultural concepts uniformly and in multiple languages. However, AGROVOC does not solve any of the access or fragmentation issues itself.

The Knowledge Graph “Agronomy Linked Data (AgroLD)” from the study by Larmande and Todorov (2021) (D2KB) is a good example of an integrated dataset. The content is FAIR-compliant and incorporates data coming from 15 different silos into a single, integrated dataset. The resulting dataset can be used to answer complex questions around plants and biology. The portal AgroLD^3^ is the main entry point to explore the Knowledge Graph. In terms of an agriculture-related portal, and with a slightly different focus, LandPortal^4^ serves integrated data about land use worldwide. The objective is to support queries around land ownership and arable land utilization.

We remark that, although vocabularies such as AGROVOC can support the creation of data portals aimed at particular needs, there is still a requirement for a more holistic approach. As outlined in the study by WEF (2023), there are a number of services needed around these portals in order to unlock their capabilities. It could also be interesting to consider creating a more flexible alternative to data portals constructed on a data aggregation approach. To achieve this aim, we propose the adoption of the recent and growing approach of the data to the agricultural domain (Joshi et al., 2021; Butte and Butte, 2022; Hooshmand et al., 2022; Bode et al., 2023; Dolhopolov et al., 2023; Goedegebuure et al., 2023; Pongpech, 2023).

## 3. The CowMesh architecture

In this section, we will introduce the CowMesh architecture by first providing a general overview of the Data Mesh approach and then explaining how this is adopted in our context. Finally, we describe the central Semantic Layer component, which is a Data Fabric, and its key role in CowMesh.

### 3.1. The data mesh

The data mesh concept was introduced by Dehghani (2022a,b) to define a set of principles for publishing data (Christ et al., 2022). From a technology point of view, a data mesh can be implemented using a variety of solutions and standards. The only particularity is the focus of application programming interface (API) to replace the (manual) handling of data dumps across systems. The novelty of the data mesh approach is not the data integration itself but rather how it is approached and considered from an organizational point of view. In particular, a data mesh is centered around four core concepts as follows:

- The preservation of **domain ownership** for the different domains may serve. As teams working together in a company system, the different data domains are expected to directly collaborate with each other and own their work—for example, in terms of data exchange or analytical work usage.
- The notion of **data products** replaces the older idea of data assets. The application of a product logic to data assets turns them into things that need to match a demand, whose value is assessed and production cost studied. Data domains may share datasets as product and/or analytical applications leveraging one or more other data products as a product of their own.
- A **data infrastructure platform** is put in place to let each data domain easily make data products available to the rest of an organization. This platform must not be limited to a particular domain and should facilitate both the creation and consumption of data products.
- An overall **federated governance** approach is applied to establish data standards and best practices to use the data mesh. This ensures the technical compatibility of all data products and can ensure compliance to rules and regulations.

It is interesting to note that these concepts describe one very well-known data publication platform, the World Wide Web. The Web features a strong notion of domain ownership. Each website publisher is responsible for its own websites and research communities publishing the outcomes of analytics on the Web, or the data within it, own those publications. Websites are by default treated as products and are routinely checked for view performances as well as optimized toward increasing those views. The web infrastructure platform based on a set of accessible software and programmatic tools make it possible for anyone to publish a new product on the Web. The W3C defines the standards and best practices that make the web run smoothly (HTTP, CSS, etc.). Finally, the Web often uses different attribution mechanisms such as Creative Common licensing^5^ and document object identifiers^6^ for attribution of content. Any new technical platform or data domain willing to join the mesh can easily do so as long as the compatibility with those standards is ensured.

### 3.2. Architecture

**Figure 1:**
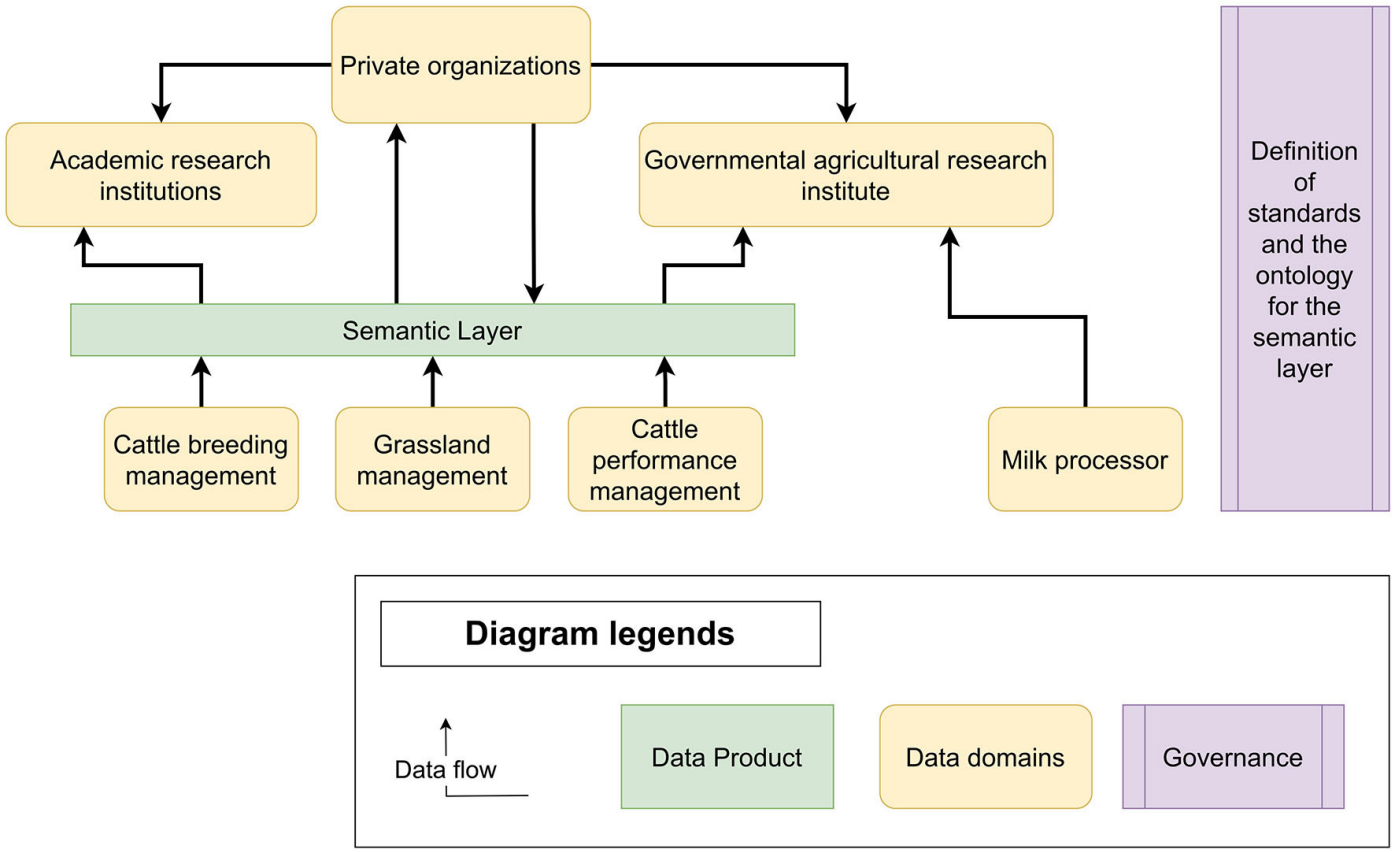
The CowMesh architecture shows the semantic layer (as a data product), several data domains, and a governance layer. A particularity of this architecture is the data product “Semantic Layer” which is a data fabric acting as a data infrastructure and interoperability layer for other data products.

To tackle the previously discussed challenges, we propose an approach based on two emerging design patterns: a data fabric and a data mesh. Whereas, these two approaches can be described as opposing each other, especially in terms of data centralization and human versus process focus, we propose to combine the two patterns so that they complement each other. Our architecture, presented in Figure 1, is composed of:

- **Data Domains** such as research institutions, public institutions, and private actors. In the Irish context, these might be the Irish Cattle Breeding Federation (ICBF),^7^ Ornua,^8^ or Teagasc.^9^ Each of these stakeholders will have some datasets and tools that they contribute as data products (not depicted in Figure 1).
- **Data Products** are contributed by data domains. Examples in the Irish context include PastureBase^10^ and the ICBF databases.^11^
- A **Semantic Layer** implemented as a data fabric is a core element of the proposed architecture and the only data product presented in Figure 1. The role of the semantic layer is to provide an integrated view over key data coming from different data domains. This does not prevent consumers of this data product from going back to the data domain sources in it—as, for example, illustrated in the link between milk processors and governmental research institutions (meaning, for instance, specific data access negotiations)—but does make it easier to consume the data.
- The **Governance** layer decides on the standards being used for the mesh overall, and the ontology driving the semantic layer.

It can be observed that the semantic layer is a data product lacking a defined data domain owning it. This is because we consider this as an open question left to specific implementations. In some cases, a research consortium might assume this role, while, in other cases, this would be one of the industrial stakeholders. For the specific Irish context, the research program VistaMilk^12^ would assume this role.

In our architecture, the semantic layer is a data fabric, created as a data product and a central part of CowMesh. Since a key objective of a data mesh is to promote decentralization, having a central semantic layer might appear to be contradictory. However, its inclusion enhances the effectiveness of the data mesh by keeping the data decentralized while connecting them at a uniform semantic and conceptual level, thus establishing interoperability. Additionally, the semantic layer sets standards and provides governance to address data privacy, ownership, and access issues.

### 3.3. Semantic layer: a data fabric

The semantic layer data product is a data fabric in our architecture, which enables interoperability among different data domains and enables governance. Although this sits at the core of the architecture, and a data fabric is a centralized approach to manage data, this does not hinder the decentralized properties of the data mesh. Both data mesh and data fabric are architectural patterns to manage data in a distributed and complex environment. The main contrasting properties of the data mesh and data fabric are as follows:

- **Scope**: A data fabric is typically designed to manage data across an entire organization, while a data mesh is more focused on specific domains, groups, or business units within an organization.
- **Approach**: A data fabric is more centralized in its approach, with a single unified architecture that connects and integrates data from different sources. On the other hand, a data mesh is more decentralized in its approach, with individual domains or teams responsible for managing their own data.
- **Governance**: A data fabric provides a centralized governance framework for managing data, while a data mesh relies on a decentralized governance model, where each domain or team is responsible for defining and enforcing its own governance policies.
- **Culture**: A data mesh is more focused on promoting a culture of data ownership and collaboration among teams, while a data fabric is more focused on standardization and consistency in the data management process.

Therefore, a data fabric is a centralized approach to managing data across an entire organization, while a data mesh is a more decentralized approach that focuses on promoting data ownership and collaboration within specific domains or business units. We use these contrasting properties to compliment each other to address the previously mentioned issues in dairy farms. The data fabric, used as a data product, establishes a uniform data model, access protocol, and governance model. On the other hand, the data mesh enables decentralized development of data products.

The role of the semantic layer in the CowMesh architecture involves:

- Providing an ontology of the concepts in the farm data that provides a uniform data model.
- Defining a set of protocols (through APIs) for accessing farm data through the concepts in the ontology.
- Integrating heterogeneous data domains in the CowMesh architecture to make them interoperable.

In summary, the semantic layer enables decentralized development in the data mesh while also providing interoperability, integration, and governance.

#### 3.3.1. Ontology

**Figure 2:**
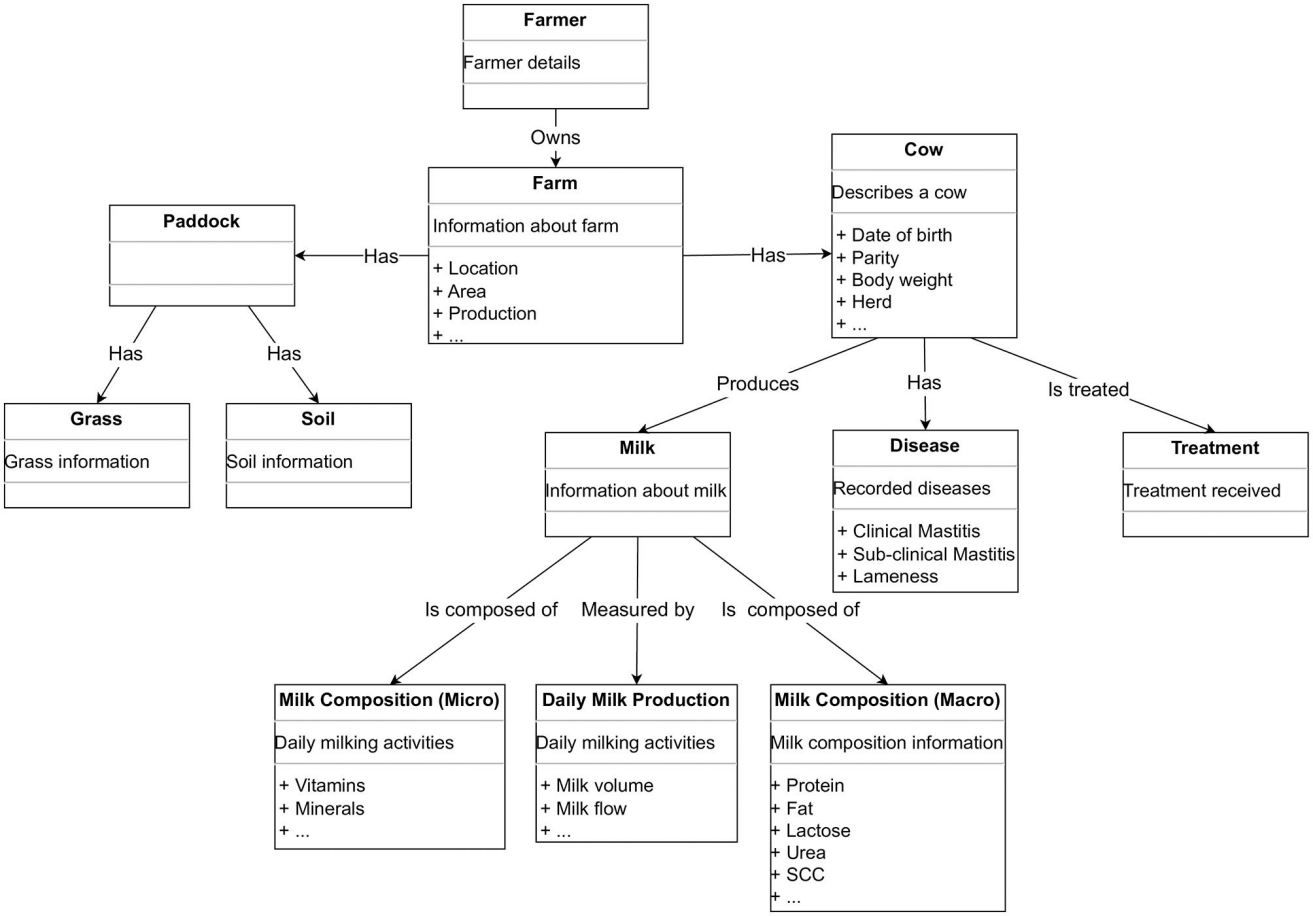
An ontology showing an example of how the concepts in the semantic layer of the CowMesh may be structured.

Like any other domain, the data in dairy farming have a set of concepts related to them. For example, all data related to cows in a single herd or a collection of dairy farms can be observed as a concept “cow.” The concept “cow,” with respect to the data, is an abstract view of a cow which is described through its properties. A cow can be described by its unique identifier, date of birth, body weight, and various other attributes. Several concepts like this—such as farm, milk, and paddock—can also be defined. Different concepts will be interconnected to describe the abstract data representation of a farm. We propose an ontology for a dairy farm, which is shown in Figure 2.

An ontology such as the one shown in Figure 2 shows an example of how dairy industry data can be structured. The different concepts in our ontology are as follows:

- Cow: Describes the cow, date of birth, etc.
- Herd: Describes properties of a herd related with Cow.
- Soil: Properties of the farm's soil.
- Grass: Properties of the farm's grass.
- Paddock: Information about a farm's paddocks related to soil and grass.
- Milk Record: milk composition including fat, protein, lactose, urea, and somatic cell count.
- Daily Milk Record: Properties for daily milking, milk yield, milk flow etc.
- Milk: Describes overall milk.
- Farm: Information about the farm, e.g., location, area, nos. of herd, and nos. of cows per herd.
- Disease Occurrence: Record of occurrences of disease in cows such as clinical mastitis, sub-clinical mastitis, lameness, and respiratory disease.
- Treatment: Treatment performed for any disease.
- Farmer: Information about the farmer.

Such an ontology sits centrally in the semantic layer, abstracting the dairy farm data to the users. Some components of the ontology might describe sensitive or personal information, such as exact farm location or farmer information. The exact data to be shared and how it is shared will be controlled by the source and governed by the semantic layer according to the agreed privacy policy. Therefore, such data instances should be ideally anonymized, or omitted based on the policy, so as to preserve data privacy (e.g., the anonymization of data in the MilkMap system described later in Section 4.2). It is important to note that the semantic layer only enables interoperability by providing an abstract model of the data (ontology) and a protocol (though an API) for data access.

#### 3.3.2. Access and access protocol

One of the main services of the semantic layer as a data product is to provide an access protocol for the farm data with respect to the provided ontology. All the data products in the CowMesh should be able to use this protocol to access data. This process can be implemented through an API, which refers to a set of protocols and tools that allows different software applications to communicate and interact with each other, thus enabling data and functionality sharing across systems. An API defines the methods and data structures that developers can use to integrate services or features into their own applications. This allows consumers to retrieve farm data in the CowMesh uniformly, even though the different data domains may have differently structured data and access protocols. The API is implemented in the semantic layer and is a data product, although the API can also reside in the source data domain, if required. For example, if a data domain is public, the data can be mapped by the semantic layer, and the API implementation can reside in the semantic layer. On the other hand, if the data domain is privately owned, the API implementation can reside as a data product with the private owner, but the access protocol and the compliance with the ontology are ensured. The particular implementation depends on the specific case. The semantic layer only provides the guidance through which it should be done, and specific implementation can be selected on a case-by-case basis.

One of the key features, here, can provide different degrees of access control and log data access, which helps keep track of the chain of custody of data. This not only makes the data access transparent but also provides an opportunity for monetization.

Another service which can be provided is to keep some commonly-used clean data in the semantic layer for easy access. This helps the products get the benefits of using pre-processed data from a central source. That said, this does not bind the data product to the limitations of the central data source, as the data products can always use the same data from the original source.

#### 3.3.3. Data product integration

Enabling access to farm data through one uniform set of protocols requires that all data domains agree on the specified ontology. One of the key functions of the semantic layer data product is to add and update new data products in the CowMesh. To enable such integration of the data products, each product needs to provide access to their data in such a way that it complies with the data ontology and the access protocol set by the semantic layer. For example, cow milk property information may be stored in two data domains, one public and one private, which are stored in different formats. To integrate these two organizations into data domains in the CowMesh, the data products should be able to access them through protocols set by the semantic layer while providing the necessary access control and privacy.

The integration of data sources can be achieved by introducing the role of a Knowledge Scientist (KS; Fletcher et al., 2020), who will perform this integration. A KS is a person who represents a bridge between the underlying data and the business requirements. In our case, a KS would communicate with the two data domains, to understand the structure of the data which they are willing to make a part of the CowMesh. The KS will have a detailed understanding of the CowMesh and the semantic layer and the required knowledge of the data source through communication. This translation can be implemented at the semantic layer end or the data product end, which can be decided on a case-by-case basis and is implementation-specific.

It is important to emphasize that the KS does not need to be an expert in the dairy industry or know specific properties about the data or data cleaning. The key role of the KS is to have basic knowledge about the concepts related to dairy industry data, knowledge about CowMesh, the ontology, and the role of the semantic layer, ability to understand requirements from communications with the data domains, and understanding the related technologies of the specific implementation.

The data sources in the CowMesh do not necessarily need to represent a large public or private organization. Smaller and independent data hosts can also participate easily in the process by following the solid protocol,^13^ guided by the protocols set by the semantic layer. A solid pod is a personal online data store that empowers users with control over their data, following the principles of the solid project for decentralized and secure data management on the web. The basic idea behind solid is to allow users to store their data in a “pod,” which is a personal online data store that they control. Users can, then, grant access to their data to apps and services as they see fit, rather than having their data silo-ed in different apps and services controlled by large corporations. Solid aims to provide a more open, decentralized, and user-controlled web, where individuals have more autonomy over their personal data and are empowered to choose which apps and services they want to use and share their data with. Therefore, instead of having full infrastructure like an organization, an individual (e.g., a farmer) with data can contribute to the CowMesh by making their data accessible as data products through solid pods, which are easy to deploy. Services such as Inrupt^14^ can be used to deploy a solid pod easily, with the assistance of a KS following the protocols set by the semantic layer.

#### 3.3.4. Governance

The governance of data, data access, and ownership is simplified by the CowMesh architecture through the semantic layer. The data domains own their data, and they decide whether to keep the data within the semantic layer or rather to keep them on their private server. Each data domain and data product can have their own governance. However, to be a part of the CowMesh, the data domains, and data products need to conform to the standards and protocols set by the semantic layer. This provides two levels of governance. The data products in the data mesh as an independent unit may have their own governance. In addition, by being a part of the CowMesh, they fall under the uniform set of protocols and standards. This streamlines the governance of the entire CowMesh. Better governance will also encourage the opening of controlled channels from private organizations through CowMesh, which can help to facilitate greater collaboration between the dairy industry and academic researchers.

One open question revolves around who will govern the CowMesh. While the answer will be specific to the context in which CowMesh is being implemented, some possibilities are as follows: (1) one of the member organizations of the CowMesh can take responsibility for governance; (2) several members of the organizations of the CowMesh can form a governance forum; (3) a neutral organization can act as a governing body. One successful example of such a governing body is The Open Subsurface Data Universe (OSDU),^15^ which regulates how oil and gas companies manage and analyze subsurface data. The goal of OSDU is to create a common data platform that allows the member companies to share and collaborate on subsurface data while also providing secure and scalable access to the data. OSDU is under the guidance of The Open Group,^16^ a global consortium that brings together industry, government, and academia to develop open standards and best practices for technology.

## 4. Use case implementations

**Figure 3:**
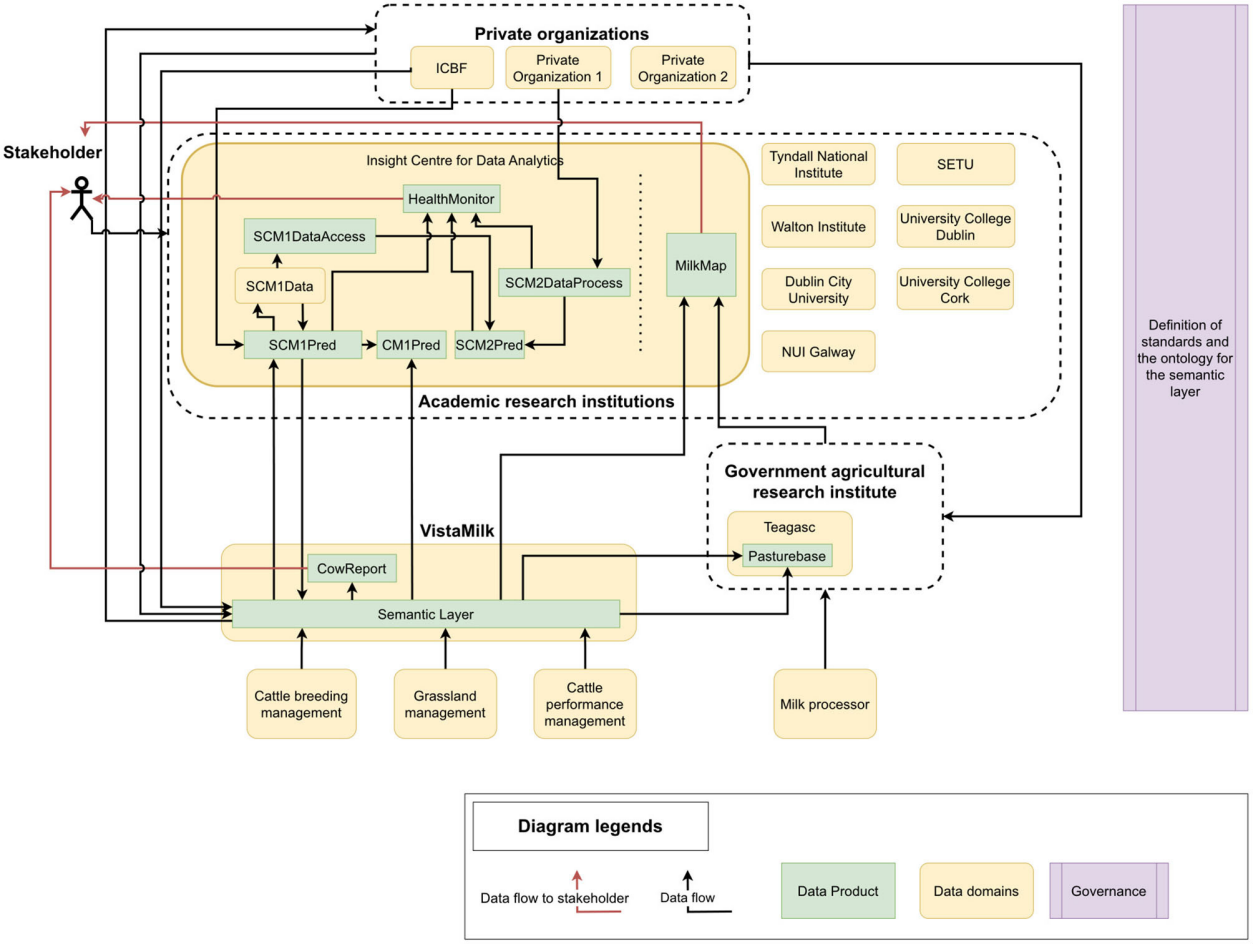
A CowMesh architecture implementation showing several data products for a specific use case.

In this section, we will present two use cases which can benefit from the proposed architecture and overcome current challenges in data-driven dairy farming. Figure 3 shows an example of CowMesh architecture in the context of the Irish agricultural sector. Here, we extend Figure 1 to demonstrate data domains and data products, together with their interactions in Ireland. In this example, the semantic layer is owned by the VistaMilk data domain, and a data product “CowReport,” which provides a periodic summary insight into the data from different sources. Such a report can directly help the farming industry analyze the data from a higher level perspective and help the stakeholders to take informed decisions.

Teagasc is the Agriculture and Food Development Authority in Ireland and has a data product PastureBase (Hanrahan et al., 2017) related to countrywide grassland management. The Irish Cattle Breeding Federation (ICBF) is a non-government organization that provides a large data repository for several areas related to dairy farms. Each academic institution or research center involved in agriculture research can be a data domain. For instance, we show some data products within the Insight Centre for Data Analytics^17^ data domain. The possible products within this domain and how they interact are described in the following subsections.

### 4.1. Mastitis prediction

Mastitis is an inflammatory response of the udder in the cow's mammary gland caused due to microorganism infections. Mastitis is divided into two types, namely, (a) clinical mastitis, where symptoms are visible to the naked eye; and (b) sub-clinical mastitis, where the symptoms are not visible but can be measured though testing. Both of the variants compromise the health and wellbeing of the cows which results in negative impact on milk production volume and quality (Halasa et al., 2007), increased veterinary costs (Cavero et al., 2007), and an increased risk of culling. Mastitis is one of the most common infections on dairy farms globally, with ~20–30% of cows in any herd likely to become infected annually (Heringstad et al., 2000). Therefore, the ability to predict the onset of clinical or sub-clinical mastitis in cows ahead of time will be of great benefit on dairy farms.

#### 4.1.1. Predicting mastitis: traditional process

Prediction of mastitis (both clinical and sub-clinical) has been previously performed using data-driven statistical and machine learning methods (Ebrahimi et al., 2019; Anglart et al., 2020; Bobbo et al., 2021). A recent study by Pakrashi et al. (2023) addressed this issue by predicting sub-clinical mastitis in Irish dairy cows up to 7 days ahead of time. Pakrashi et al. (2023) use machine learning algorithms to train prediction models using the data from seven Irish research farms spanning 9 years of data, which consisted of the following information:

- Daily milk yield and other milking information;
- Milk composition (e.g., fat, lactose, protein, and somatic cell count)
- General cow features available at the farm (genetic information of the cow, how many times the cow has given birth, etc.)
- Other derived variables from the above information (e.g., how many times a cow has been diagnosed before, if a cow was treated before, mean, and standard deviation of the change in milk composition in the last 7 and 15 days)

The final delivery of the data for the study conducted by Pakrashi et al. (2023) was in the form of CSV^18^ files sent *via* email. The general cow features were included in one CSV file, and milk composition data in another CSV file. Each of the CSV files required special attention for data cleaning, and then, they were joined to make the dataset required. In addition, for the specific task in hand, several derived variables were created for the project, which was not a part of the provided data. This was, then, analyzed, and a machine learning model was trained. While the development was performed, a new batch of data from a subsequent year was available and transferred through email in a similar set of CSV files, which was again combined after fixing a few issues due to incompatibilities with the previous data received.

The above process, if considered in isolation, is relatively straightforward. However, deploying the pilot project in a practical real-world scenario presents a number of problems as follows:

1. As farms generate new data, accessing data *via* email or through a single central data repository is cumbersome.
2. Different teams working on the data coming from the same source had different variable names assigned by the corresponding research teams. There was no clear data description, therefore it was hard to communicate between the teams about the problems.
3. The same data cleaning and transformation were performed independently by the different teams using the same data, leading to redundancy and duplication of effort.
4. The final training dataset prepared by Pakrashi et al. (2023) was found to be useful to other teams. However, processed datasets were sent as a zipped set of CSV files *via* email. This data generation and processing consume a significant amount of time. Additionally, when data are sent *via* email, any updates or changes in processing or data require resending the files, which are often overlooked, leading to continued use of outdated data by other teams.
5. Different teams worked on predicting mastitis through different approaches (Jin et al., 2023). The main target is to predict mastitis in cows using different data available from a dairy farm. Therefore, these projects and the predictive models can be combined to complement each other to build a better mastitis predictor. However, without a shared framework, it is extremely difficult to integrate the products coming from different frameworks.
6. The exact details around data ownership were not clearly defined.

The above issues show that, although valuable data were available from a central source, the processing and analysis work were scattered across silo-ed teams, and the products the teams built (mastitis predictor) were also bound within the team. Aligning with the major challenges defined by WEF (see Section 2), point 1 is a fragmentation issue, while points 2, 3, 5, and 6 relate to standardization. The problems mentioned in points 1, 4, and 5 represent access issues.

#### 4.1.2. Mastitis predictor as a data product in CowMesh

Our proposed data mesh architecture helps address these issues. To demonstrate this, we describe how a collection of projects related to mastitis research can be integrated into the data mesh in an Irish context.

First, the required data mentioned previously (e.g., daily milk data and milk composition data) are aligned with an ontology defined by the semantic layer. This is performed with the help of the data product team and the KS. Such an ontology is shown in Figure 2.

Figure 3 shows how the set of mastitis-related products could be incorporated into the CowMesh. The data domain is named “SCM1_Data” (SCM stands for sub-clinical mastitis), and the six data products are named “SCM1Pred,” “SCM1DataAccess,” “SCM2Pred,” “SCM2DataProcess,” “CM1Pred” (CM stands for clinical mastitis), and “HealthMonitor.” The roles of these products are explained below.

The “SCM1Pred” product is developed by a research group which is focused on predicting sub-clinical mastitis from daily milking information and historical data about milk and cows. This data product consumes data through protocols set by the semantic layer, by using the API, following the ontology. Therefore, given the ontology (such as Figure 2), this domain will be mainly working with the concepts, such as “Cow,” “Milk,” and “Disease.” The outputs of this product are the predictions and the trained machine learning model. Such a product sharing the outputs of the predictions can be incorporated into a report such as the “CowReport” data product or directly sent to the farmer to assist in decision-making. In addition, potentially the trained machine learning model may be required by other research teams so that they can build another product on top of this (e.g., explaining the predicted outcome). Some additional variables were developed in this project, which were found to be useful to other teams. Therefore, the data product “SCM1DataAccess” provides an API through which the additional variables are accessible.

The data product “CM1Pred” is developed by a research team which works on predicting clinical mastitis. This product accesses data through the semantic layer and uses the additional variables through “SCM1DataAccess,” as well as the predictions from “SCM_Pred” as required. Here, “CM1Pred” does not have to recompute the additional variables or receive the variables through cumbersome CSV files.

On the other hand, “SCM2Pred” is a data product which performs sub-clinical mastitis prediction but uses a different perspective and a different set of variables. In this project, some of the data, “SCM2Pred,” which are owned privately and are not allowed to be kept. The data pre-processing logic is provided as a service through the “SCM2DataProcess” data product. Therefore, this product can be accessed to use the same data pre-processing while accessing the actual data through the semantic layer from the same sources. The access and sharing of the data, which might be privately held, are governed by the semantic layer. The sharing protocol and other agreements would have been already done while integrating the related data domains.

The data product “HealthMonitor” can be a dashboard summarizing the cows' health in the farms. This takes data from the semantic layer, summarizing the outputs of “CM1Pred,” “SCM1Pred,” and “SCM2Pred.”

The results of these products are also consumed by the report generation product of the semantic layer data domain, such as “CowReport.” Including such information in the report can provide dairy farmers and stakeholders with a high-level picture of the health of large herds. Additionally, incorporating mastitis prediction information into the report can prevent farm losses, address cattle welfare, and improve milk production by enabling farmers to take preventive measures ahead of time, before a cow shows the signs of clinical or sub-clinical mastitis.

Issues 1, 2, and 3 are addressed by the ontology and the uniform API provided by the semantic layer and central cleaned accessible data kept with the semantic layer. Issues 4 and 5 are addressed by the data products “SCM1DataAccess” and “SCM1Pred,” respectively, which enable the use of the data sharing. These data products, like any other data products in the mesh, would also adhere to the semantic layer ontology.

As each of the data domains is responsible for managing their data by adhering to the semantic layer ontology, cleaning, pre-processing, generating derived attributes, and keeping them updated, the data quality issue is addressed. The access of the data is limited through the API and governed by the semantic layer. Therefore, access can be controlled and the chain of ownership of the data can be tracked, resulting in better data transparency and clear ownership boundaries, thus also addressing issue 6.

To be a part of the CowMesh, it is necessary to interact with the KS to ensure compliance with the protocols. The KS, working with the corresponding products, will assist the relevant team to become integrated into the CowMesh.

### 4.2. MilkMap

Ireland has a seasonal milk supply influenced by changing weather, soil nutrition, and a host of other environmental and societal factors. In addition to milk yield, the primary change in milk across a season is its composition, i.e., variation in macro (protein, fat, and lactose) and micro (minerals, vitamins, and other bio-actives) constituents. Milk composition determines the yield of dairy products produced by a farm or processor, which can be logistically complex given the fractionation, fermentation, and preservation techniques, and end applications being employed across the sector. Moreover, the compositional makeup of milk determines its functionality, processability, and ultimately its final end use (i.e., as a consumer food or ingredient in another food). For example, consistent manufacture of milk gel-based products such as cheese, casein, or yogurt is highly dependent on the protein and mineral composition of milk. Another example is the relationship between nutrient composition (i.e., protein, minerals, and other ionic species) and heat stability of milk.

#### 4.2.1. MilkMap: traditional process

The MilkMap system is designed to visually represent dairy processing in Ireland. This specialized tool necessitates significant custom processing of the aforementioned raw dairy values. This mapping application is designed to provide additional analytical capabilities in an agricultural context, including the monitoring of dairy production and the provision of time series forecasting (e.g., for yield and composition). While the architecture employed in the MilkMap system considers generalized geographical patterns over long period of time, it also provides the means to drill down into specific localized regions to explore the trends and patterns specific to each region. The final mapping application is ultimately made available to stakeholders across the organization, allowing them to leverage the full potential of the data originally collected. The processed data used in the mapping project are also available in a form that can easily be employed in different systems within the organization.

The delivery of this specialized application for dairy processing in Ireland necessitates significant custom processing of the aforementioned raw dairy values. For instance, the anonymization of dairy data was achieved through transformation of latitude and longitude coordinates to lower resolution H3 hexagonal (Brodsky, 2018) values. These data were transferred from dairy processors to a central repository, allowing for a distributed utilization of this information in application development. No sensitive information is available to either the MilkMap application or the data processing of dairy values needed for it. Other data sources could also be potentially integrated into the MilkMap system. For instance, mid-infra-red (MIR) data taken from existing instruments located across processing plants in Ireland, and grass growth data retrieved from a source such as PastureBase (Hanrahan et al., 2017).

During the development of the MilkMap, several challenges were encountered as follows:

1. Obtaining access to the data was a major hurdle, particularly from the dairy cooperatives, due to legal and privacy concerns. Extensive negotiations and agreements were required to obtain access to the information necessary for the successful implementation of the MilkMap.
2. Combining data from multiple sources was complex and time-consuming, as each source presented its own obstacles to access. Updating and accessing the data were also problematic and necessitated a special Secure File Transfer Protocol (SFTP) connection set up in each case.
3. Noisy data, missing values, and a lack of standardization in data formats presented additional issues, as did the inability to directly communicate with dairy farms or cooperatives, with interaction predominantly passing through the ICBF. Outlier detection was necessary to handle anomalous values, however, pinpointing their root cause proved difficult due to the involvement of numerous intermediaries in the data sharing pipeline.
4. Privacy concerns limited the specificity of the data, making it infeasible to drill down to the level of individual farms when performing detailed analysis.

#### 4.2.2. MilkMap as a data product in CowMesh

Overcoming these challenges required careful consideration of issues around data cleaning, standardization, governance, and security measures to ensure the accuracy and completeness of the data. To reduce the overhead in terms of time and effort, we propose that the MilkMap system could be implemented as a data product within the data mesh, which would help mitigate the issues listed above. The specific benefits of this approach are as follows:

1. To clarify data access issues, such as those around privacy and legal requirements, the semantic layer would prove useful. Since CowMesh provides increased trust and transparency around data usage, we believe that the data sources are more likely to join the mesh and supply the required data.
2. The issue of cumbersome processing of data coming from different sources with different access protocols and data structures can be addressed by the uniform data ontology and data access protocols set by the semantic layer.
3. The issue around noisy and inconsistent data can be handled at the semantic layer when integrating the data domain into the CowMesh. By conforming to the semantic layer protocols, consistent practices for data cleaning, and standardization can be enforced. In addition, since the data domain must now adhere to the data ontology, each feature in the data will be documented (e.g., in terms of range and relationship to other features).
4. The privacy and anonymization issues can be handled at the semantic layer or at the corresponding data domain. In either case, the data handling pipeline can be organized such that data products only have access to a subset of data that is necessary and sufficient as controlled by the data domain.

## 5. Advantages and opportunities for CowMesh

The objective of the CowMesh is to add value to the dairy industry through intelligent data processing. To do this, the products need access to clean data from the farms to act upon. CowMesh provides the following main advantages which encourage farms and data sources to share data, such that products can provide analytical and predictive insights, which adds value to the dairy farms.

- **Trust**: Data domains and data products can rely on the accuracy and quality of the data they are working with, as well as the security of the data and the trustworthiness of the data sources. By establishing trust in the data and the data sources, organizations can make better decisions and extract more value from the data.
- **Privacy and transparency**: By using the decentralized property of the data mesh, data can be privately held. The nature of the CowMesh allows the private data domains to decide how much data they want to make accessible to the CowMesh. In addition, the chain of ownership can be tracked and audited, ensuring transparency and compliance.
- **FAIR data**: FAIR refers to **F**indable, **A**ccessible, **I**nteroperable, and **R**eusable. By ensuring that data follow the data ontology and the access is standardized through the set of access protocols and interfaces (e.g., well-defined APIs), organizations can make it easier for users to discover and access relevant data, as well as combine and analyze data from multiple sources. This can lead to more accurate insights and better decision-making.
- **Decentralized interoperability**: CowMesh combines the contrasting characteristics of data mesh and data fabric and enables data and products to be decentralized while providing one central protocol to be followed, therefore enabling interoperability.
- **Governance**: Governance becomes easier, as the CowMesh follows the standards and protocols set by the semantic layer. This makes the different products follow the standards and protocols easily.

These points address the three main challenges (fragmentation, standards, and access) defined by WEF in the agricultural domain as mentioned in Section 2. Trust, Privacy and Transparency, and Governance can bring cultural changes with respect to how data are shared, which can encourage more organizations and individuals to share their data, addressing the challenge of “standards,” whereas FAIR Data and Decentralized Interoperability address the challenges of “fragmentation” and “access” issues.

Several resources and infrastructures need to be maintained to run the CowMesh including designing the specific architecture, hiring a KS, maintaining the APIs and the semantic layer, and maintaining security of the systems and servers. This would require some funds to be spent on CowMesh. As CowMesh is a service which the data domains and data products will use; several aspects of the CowMesh can be monetized, which can help maintain the framework. In addition, through monetization, the individual owners of data (e.g., farmers) and the larger organizations can benefit by charging for the data in an on-demand fashion. This monetary incentive may help more data sources to contribute to CowMesh and get compensated for their data while keeping the data ownership to the corresponding source and maintaining transparency. Some of the ways in which CowMesh can be monetized are as follows:

- **Data use**: The data are accessed through the API, and the use is tracked by the semantic layer. Using this, the total data used by each data product can be tracked and charged. This charge can, then, be distributed to the farmers and the institutions generating the data.
- **Data access**: The uniform data access, interoperability, and also possibly cleaned data are provided by the semantic layer of the CowMesh. Therefore, this service provided by the semantic layer can be monetized. For example, the access to the API calls (not the data) as well as the integration to the CowMesh can be monetized.
- **Report or dashboard consultation**: The semantic layer can generate a periodic report data insights and the analytical components of the CowMesh, for which the organizations and the farmers can pay. This can be a direct value added to the farming industry to see the higher level picture and enable the farmers and the organizations to take updated and informed decisions.
- **Predictive analytics**: Advanced products, such as predicting mastitis, lameness, or ketosis in cows, can be treated as add-on services, which can be offered to farmers on a subscription basis. This is a direct benefit to the farm, as these predictive data products consume data from the farms and then feed back predictive insights to help the farmers.

While the directions above indicate the potential opportunities that the CowMesh can bring, a detailed analysis of the monetization of the CowMesh is outside the scope of the current study.

The different consumers of CowMesh include farmers, researchers, commercial dairy organizations, and veterinary institutions. Farmers can contribute data to CowMesh through data products or the semantic layer. Research organizations, veterinary institutions, and commercial dairy organizations execute specific projects aligned with dairy industry needs and farmers' requirements. Project results benefit farmers through reports and other data products. Research findings can also be shared with commercial dairy organizations for commercialization or as reports. Each party can seamlessly integrate *via* CowMesh, retaining data ownership and transparency of usage, while benefiting from services and products offered, contributing to the improvement of the dairy industry at a national level.

## 6. Discussion

This study presents a data architecture, CowMesh, designed to unify disparate dairy industry data under a uniform, interoperable, and decentralized framework, thus enabling the products using the data to create value for the dairy farmers and the dairy industry. CowMesh is a combination of data mesh and data fabric. The data mesh's functionality helps the different data products using the dairy data to operate in a manner that is independent and decentralized. The central data product semantic layer is a data fabric, which provides a single unified data model and protocol. This enables connection to and integration of data from different sources within the data mesh. This enables uniform governance, creates trust, promotes data transparency, and keeps the data and the data products decentralized while providing interoperability within the data products. In future, a similar framework can be developed for other agricultural industries tailored for their specific requirements. In addition, specific details about the governance and semantic layer can be explored in an Irish context. Finally, a pilot project to implement CowMesh should be explored in future.

## Data availability statement

The original contributions presented in the study are included in the article/supplementary material, further inquiries can be directed to the corresponding author.

## Author contributions

CG conceptualized the architecture. AP and BM enhanced the architecture and specific cases. AP, CG, and BM contributed to conception and the design of the architecture and designed the mastitis prediction case study. DW and DG designed and wrote the MilkMap case study. AP wrote and finalized the manuscript. All authors contributed to manuscript revision, read, and approved the submitted version.

## Footnotes

^1^https://www.w3.org/TR/dwbp/ (visited August 17, 2023).

^2^https://agrovoc.fao.org/browse/agrovoc/en/ (visited August 17, 2023).

^3^http://agrold.org (visited August 17, 2023).

^4^https://landportal.org/ (visited August 17, 2023).

^5^https://creativecommons.org/licenses/ (visited August 17, 2023).

^6^https://www.doi.org/ (visited August 17, 2023).

^7^https://www.icbf.com/ (visited August 17, 2023).

^8^https://www.ornua.com/ (visited August 17, 2023).

^9^https://www.teagasc.ie/ (visited August 17, 2023).

^10^https://pasturebase.teagasc.ie (visited August 17, 2023).

^11^https://www.icbf.com/the-icbf-database/ (visited August 17, 2023).

^12^https://www.vistamilk.ie/ (visited August 17, 2023).

^13^https://solidproject.org/ (visited August 17, 2023).

^14^https://www.inrupt.com/ (visited August 17, 2023).

^15^https://osduforum.org/ (visited August 17, 2023).

^16^https://www.opengroup.org/ (visited August 17, 2023).

^17^https://www.insight-centre.org/ (visited September 19, 2023).

^18^Comma Separated Value file format.
